# Field Trial Demonstrates Superiority of a Live Attenuated Vaccine for Integrated Control of *Mycoplasmal pneumonia* of Swine and Improved Production Performance in Commercial Swine Farms

**DOI:** 10.3390/ani16142252

**Published:** 2026-07-21

**Authors:** Changhua Lin, Yifei Xiang, Jiaxia Jiang, Yangzu Zhang, Hao Wang, Jiakang He

**Affiliations:** 1 Guangxi Key Laboratory of Animal Breeding, Disease Control and Prevention, Guangxi Zhuang Autonomous Region Engineering Research Center of Veterinary Biologics, College of Animal Science and Technology, Guangxi University, Nanning 530004, China; 2318493015@st.gxu.edu.cn (C.L.); xiangyifei003@163.com (Y.X.);; 2 Guangxi State Farms Yongxin Animal Husbandry Group Co., Ltd., Nanning 530022, China; 3 School of Agricultural Engineering, Guangxi Polytechnic University, Nanning 530226, China

**Keywords:** *Mycoplasma hyopneumoniae*, live attenuated vaccine, inactivated vaccine, field trial

## Abstract

A field trial was conducted on a single commercial swine farm to compare the integrated control efficacy of an inactivated vaccine and a live attenuated vaccine against mycoplasmal pneumonia of swine (MPS). Mhp exposure in the herd was confirmed by qPCR prior to trial initiation. Initially, 30 five-day-old piglets were randomly assigned to three groups (10 piglets per group): non-immunized control, inactivated vaccine, and live attenuated vaccine, for clinical safety evaluation. Based on this, another 900 five-day-old piglets, confirmed Mhp-negative by antigen detection, were randomly allocated to three separate rearing zones (300 piglets per zone), with each zone randomly assigned to one of the three treatment groups. Parameters assessed included mucosal (sIgA) and serum (IgG) antibody levels, pulmonary inflammatory cytokine (e.g., IL-1β, IL-6, TNF-α) expression, lung lesion scores, and histopathological changes, with all evaluations performed by technicians blinded to group allocation. Growth performance and economic indicators were also recorded. Both vaccines exhibited favorable safety profiles. The live attenuated vaccine significantly enhanced nasal sIgA and serum IgG levels (*p* ≤ 0.01), whereas the inactivated vaccine only significantly elevated serum IgG (*p* ≤ 0.01) but had no significant effect on sIgA (*p* ≥ 0.05). The live attenuated vaccine group showed the lowest lung lesion scores, as well as the mildest pulmonary inflammation and pathological damage. Meanwhile, swine in this group achieved higher average daily gain, lower mortality, and lower feed conversion ratio, with a 3-day shorter rearing period than the non-immunized group and an 11.27 CNY reduction in combined vaccination and medication cost per swine compared with the inactivated vaccine group. In conclusion, under the conditions of this single-farm trial, the live attenuated vaccine demonstrated superior efficacy in integrated MPS control and production performance improvement.

## Simple Summary

Pneumonia caused by *Mycoplasma hyopneumoniae* is a common and economically damaging disease in swine farms worldwide. Vaccination is the main control measure, but field performance of different vaccine types remains unclear. A large-scale field trial was conducted on a commercial swine farm in Guangxi to compare a live attenuated vaccine with an inactivated vaccine. The results show that the live attenuated vaccine provides better immune protection, reduces lung damage, improves production performance, and lowers farming costs. These findings support the wider use of live attenuated vaccines in large swine farms, helping to promote healthier and more efficient swine production.

## 1. Introduction

*Mycoplasma hyopneumoniae* (Mhp) is the primary etiological agent of mycoplasmal pneumonia of swine (MPS) [1,2], also known as swine enzootic pneumonia [3,4]. Mhp infection is characterized by chronic, non-fatal respiratory signs, including coughing, dyspnea, growth delay, and reduced feed conversion efficiency [1,5]. Although associated with low mortality, Mhp infection causes marked growth impairment and reduced production performance in swine herds, resulting in substantial economic losses for the global swine industry [6]. Mhp establishes infection by adhering to the ciliated epithelial cells of the respiratory tract [7], severely compromising the mucociliary clearance function of the airway mucosa [4,8], thereby exacerbating secondary infections with other pathogens, such as porcine reproductive and respiratory syndrome virus (PRRSV) and porcine circovirus type 2 (PCV2) [2,8]. A recent surveillance study of 1362 porcine lung samples from 14 prefectures and cities in Guangxi reported an Mhp positivity rate of 48.09% [9], highlighting the urgent need for effective control and eradication of Mhp in this region.

Vaccination is well established as the primary strategy for MPS control [10]. Currently, two main types of MPS vaccines are commercially available: inactivated vaccines and live attenuated vaccines [4]. Inactivated vaccines exhibit high safety and ease of storage, acting primarily by inducing humoral immune responses [11]. In contrast, live attenuated vaccines can activate stronger cellular and mucosal immune responses by mimicking natural infection routes [11]. Theoretically, mucosal immunity plays a critical role in defending against pathogens that invade via the respiratory tract [12,13]. Secretory immunoglobulin A (sIgA) is the major effector molecule of local respiratory mucosal immunity [13] and can effectively prevent Mhp from adhering to and colonizing the respiratory mucosa. Furthermore, Mhp infection induces a persistent pulmonary inflammatory response [4,14], characterized by elevated levels of pro-inflammatory cytokines, such as IL-1β, IL-6, and TNF-α [4,14], which in turn exacerbates lung tissue damage [14,15].

Although previous studies have evaluated the immune efficacy of the MPS live attenuated vaccine (strain 168) in the Chinese native Ningxiang pig breed [16], confirming its applicability to local native breeds, the evaluation of immune efficacy in commercial crossbred pig populations remains lacking, and systematic studies on histopathological examination, inflammatory cytokine detection, and production benefit analysis are also absent. Most existing studies have assessed vaccine immunogenicity or single protective parameters under laboratory conditions [17], overlooking the integrated impact of vaccination on overall herd health, the respiratory inflammatory microenvironment, and farm economic returns [18]. An ideal vaccine should not only induce protective immunity but also effectively modulate inflammation, alleviate lung injury, and improve production performance and economic indicators.

In this study, a field trial was conducted under real production conditions in a large-scale swine farm to systematically compare the inactivated and live attenuated Mhp vaccines with respect to clinical safety, mucosal and humoral immune responses, pro-inflammatory cytokine levels, gross lung lesions and histopathological scores, production performance, and economic benefits. The findings are intended to provide a robust experimental basis for vaccine selection and use on swine farms, and to contribute a superior immunization strategy for the control of MPS.

## 2. Materials and Methods

### 2.1. Experimental Site and Management Practices

This study was conducted in a large-scale swine farm with an inventory of over 10,000 heads in Guangxi, China. Normal production operations of the farm were maintained throughout the trial period. Routine health care and immunization procedures were carried out, and no additional vaccines or drugs targeting Mhp or related pathogens were supplemented. The experimental swine were housed in barns with consistent environmental conditions and management regimes. All swine were provided with automatic drinkers and feeders, allowing ad libitum access to nutritionally balanced diets and clean water. The temperature and humidity of the housing environment were regulated by a fully automated control system to meet the physiological requirements of the swine throughout the trial period, and animal welfare was ensured.

### 2.2. Vaccines and Immunization Routes

Live attenuated vaccine: Zhibining^®^ live MPS vaccine (strain 168, supplied by ZF-Hua Biotechnology Co., Ltd., Nanjing, Jiangsu, China) was administered via intrapulmonary injection at 7 days of age, according to the manufacturer’s recommended protocol. Inactivated vaccine: A commercial inactivated Mhp vaccine (strain J) was administered via intramuscular injection at 14 days of age, also following the manufacturer’s recommended protocol.

### 2.3. Experimental Grouping and Treatment of Piglets

Trial 1: Vaccine safety evaluation. Thirty healthy 5-day-old piglets (Duroc × Landrace × Yorkshire, half male and half female) were randomly selected. After confirmation of Mhp antigen-negative status, the piglets were randomly divided into three groups (10 per group): non-immunized control group, inactivated vaccine group, and live attenuated vaccine group. All piglets were individually identified with ear tags.

Trial 2: Comparative field trial of vaccines. A total of 900 healthy 5-day-old piglets (Duroc × Landrace × Yorkshire, half male and half female) were randomly selected from the same batch. After confirmation of Mhp antigen-negative status, the piglets were allocated to three separate rearing zones, with each zone serving as an independent experimental unit (300 piglets per zone). Zones were randomly assigned to one of three treatments: non-immunized control, inactivated vaccine, or live attenuated vaccine. All piglets were individually identified with ear tags. All piglets were vaccinated according to the immunization schedule described above.

### 2.4. Mhp-Specific DNA Detection

Swine herds (total 3500 heads) originating from the same production region and not vaccinated against Mhp were selected. Following a previously described method [19], nasal and throat swab samples were randomly collected from 10% of the swine (*n* = 350) using sterile cotton swabs. The swabs were placed in sterile centrifuge tubes containing 1 mL of physiological saline and vortexed. Total DNA was extracted using a DNA extraction kit (Sangon Biotech (Shanghai) Co., Ltd., Shanghai, China). Mhp DNA detection was performed by qPCR using Mhp-specific primers targeting the P97 cilium adhesin gene [Gene Accession number U50901] (primer sequences are shown in Table 1). The amplification protocol was as follows: initial denaturation at 94 °C for 120 s, followed by 30 cycles of denaturation at 94 °C for 30 s, annealing at 57 °C for 30 s, and extension at 72 °C for 60 s, with a final extension at 72 °C for 420 s. Samples with a Ct value < 30 were defined as Mhp antigen-positive.

### 2.5. Vaccine Safety Evaluation

The clinical safety of the inactivated and live attenuated MPS vaccines was assessed using the non-vaccinated control group as a reference. Clinical observations were performed daily for 7 consecutive days post-vaccination, and safety was quantitatively analyzed using a standardized scoring system (Table 2). Higher scores indicated better vaccine safety.

### 2.6. Detection of Mhp-Specific sIgA Antibodies

Nasal swab samples were randomly collected from the experimental swine (*n* = 60) prior to slaughter. sIgA levels were measured using an Mhp ELISA antibody (sIgA) detection kit (Jiangsu Academy of Agricultural Sciences, Institute of Veterinary Medicine, Nanjing, China), which effectively distinguishes antibodies induced by field infection from those induced by vaccination [20]. The assay was performed according to the manufacturer‘s instructions, and results were expressed as S/P values. According to the kit criteria, S/P < 0.15 was considered negative, S/P > 0.20 positive, and S/P between 0.15 and 0.20 suspect.

### 2.7. Detection of Mhp-Specific IgG Antibodies

Blood samples were randomly collected from the experimental swine (*n* = 60) prior to slaughter. The blood samples were allowed to stand at 4 °C for 2 h, then centrifuged at 3000 r/min for 10 min to separate the serum. The serum was aliquoted and stored at −80 °C until use. Mhp-specific IgG antibody levels in the serum were measured using an Mhp ELISA antibody (IgG) detection kit (AffiGEN Inc., Vancouver, BC, Canada), following the manufacturer’s instructions. Results were expressed as S/P values. According to the kit criteria, S/P > 0.4 was considered positive.

### 2.8. Detection of Inflammatory Cytokines in Bronchoalveolar Lavage Fluid (BALF)

Fresh isolated lungs were randomly selected from each group (*n* = 30) at slaughter. Using a syringe, 50 mL of sterile PBS was slowly infused through the tracheal opening, and the lungs were gently pressed for 2 min to lavage. The BALF was filtered through sterile gauze into sterile centrifuge tubes and centrifuged at 5000 r/min for 10 min at 4 °C, and the supernatant was collected. The expression levels of IL-1β, IL-6, and TNF-α in BALF were determined using commercial ELISA kits (Shanghai Enzyme-linked Biotechnology Co., Ltd., Shanghai, China).

### 2.9. Lung Scoring and Histology

#### 2.9.1. Lung Lesion Scoring

**Figure 1:**
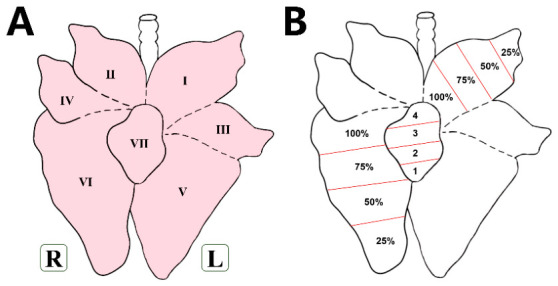
Schematic diagram of lung scoring. (**A**): Diagram of lung lobes (I: left apical lobe; II: right apical lobe; III: left cardiac lobe; IV: right cardiac lobe; V: left diaphragmatic lobe; VI: right diaphragmatic lobe; VII: accessory lobe). (**B**): Detailed scoring criteria for a single lung lobe.

Lungs (*n* = 30) were fully removed by slaughterhouse technicians unaware of group allocation. The lesions on each lung lobe were observed and photographed (both dorsal and ventral surfaces). A 28-point scoring system was adopted [21], whereby the infected area of each lobe was classified into five categories (Table 3) and each lobe was scored accordingly (see Figure 1A,B). The scores of the seven lung lobes were summed.

#### 2.9.2. Pulmonary Histopathology (Hematoxylin and Eosin Staining)

Isolated lungs were fixed in 4% paraformaldehyde (PFA) for 48 h. After appropriate trimming, the tissue samples were rinsed with running water, dehydrated using a graded ethanol series, and embedded in paraffin blocks. The paraffin-embedded tissue blocks were sectioned at 3 μm and baked at 65 °C for 4.5 h, followed by deparaffinization and rehydration. Finally, the sections were stained with hematoxylin and eosin (H&E) and coverslipped. Lung sections were examined in a blinded manner and assigned severity scores according to the criteria in Table 4.

### 2.10. Production Performance and Economic Indicators

During the trial period, dedicated farm technicians recorded the initial body weight, sale weight, daily feed consumption, mortality and culling rates, and costs of drugs and vaccines for each group. These data were used to systematically evaluate the impact of different immunization protocols on swine growth performance and farm economic returns. The relevant indicators were calculated using the following formulas:

(1) Average sale weight = Total sale weight/Number of swine sold
(2) Average daily gain = Total weight gain/(Number of experimental swine × rearing days)
(3) Mortality rate = (Number of dead swine/Total number of experimental swine) × 100%
(4) Feed conversion ratio = Total feed consumption/Total weight gain
(5) Cost of vaccines and drugs per swine = Total cost of vaccines and drugs/Total number of experimental swine

### 2.11. Statistical Analysis

All data were provided by the veterinarians working on the farm. Basic data collation was performed using Microsoft Excel. Data are presented as mean ± standard deviation (SD). One-way analysis of variance (ANOVA) with least significant difference (LSD) post hoc test was used to compare the results between groups. Statistical analyses and graphing were performed using the GraphPad Prism 10 software. A *p* < 0.05 was considered statistically significant, and *p* < 0.01 was considered highly significant.

## 3. Results

### 3.1. Mhp Positivity Rate in Swine Herds Before Immunization

The Mhp positivity rate was assessed using the Mhp-specific qPCR in one production zone of the swine farming company. The test results (Table 5) showed an Mhp positivity rate as high as 64%, indicating a high Mhp infection pressure in this area. Furthermore, the detection results from nasal swabs and throat swabs were consistent; therefore, nasal swabs were used for sampling and detection in subsequent studies.

### 3.2. Vaccine Safety

**Figure 2:**
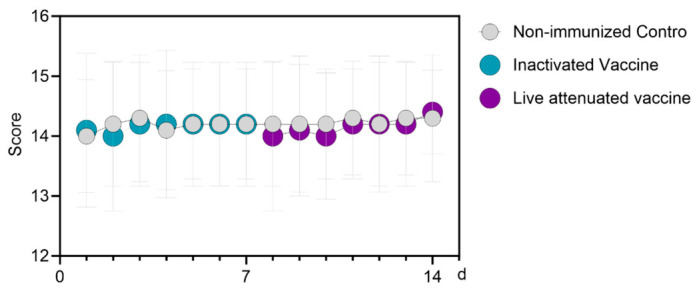
Vaccine safety scores after immunization.

The preliminary clinical safety evaluation over a 7-day period (Figure 2) showed that, compared with the non-vaccinated control group, neither the inactivated vaccine group nor the live attenuated vaccine group exhibited any obvious adverse reactions, indicating that both the inactivated and live attenuated MPS vaccines have a favorable safety profile.

### 3.3. Effects of Different Immunization Protocols on sIgA and IgG Antibody Levels

**Figure 3:**
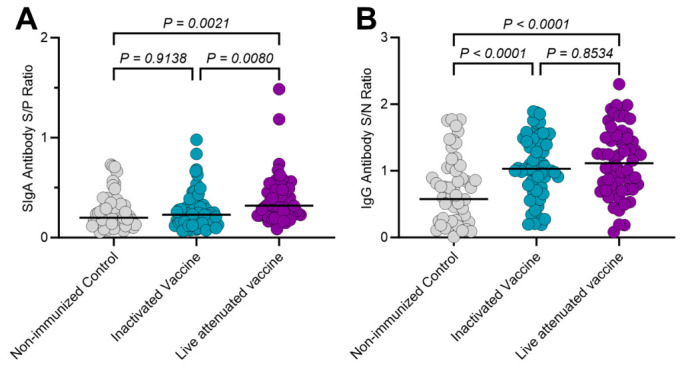
Detection of sIgA and IgG antibody levels. (**A**): Specific sIgA antibody levels in nasal swabs; (**B**): Specific IgG antibody levels in serum.

sIgA is the main effector molecule of respiratory mucosal immunity and directly reflects the local protective immune response induced by vaccination. The results (Figure 3A) showed that the sIgA antibody level in nasal swabs of the live attenuated vaccine group was significantly higher than that in the non-vaccinated control group and the inactivated vaccine group (*p* < 0.01), whereas no significant difference was observed between the inactivated vaccine group and the non-vaccinated control group (*p* > 0.05).

Serum IgG antibody level is an important indicator of systemic humoral immune responses and reflects the systemic protective immunity induced by vaccination. The serum IgG antibody results (Figure 3B) showed that serum IgG antibody levels in both the inactivated vaccine group and the live attenuated vaccine group were significantly higher than that in the non-vaccinated control group (*p* < 0.01), with the live attenuated vaccine group showing higher IgG levels.

These results indicate that the Mhp live attenuated vaccine induces both strong mucosal immunity and systemic humoral immunity, whereas the inactivated vaccine mainly induces systemic humoral immunity and has a limited effect on mucosal immunity.

### 3.4. Effects of Different Immunization Protocols on Inflammatory Cytokine Levels in BALF

**Figure 4:**
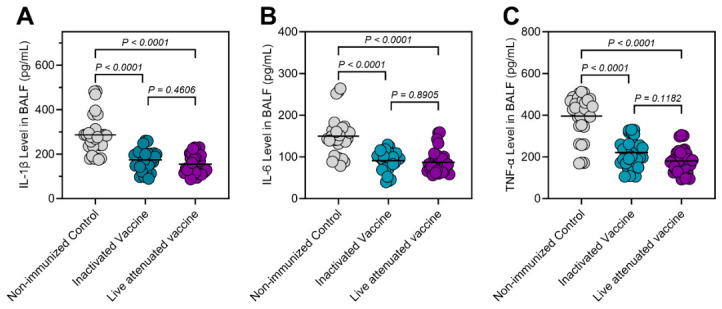
Inflammatory cytokine levels in BALF. (**A**): IL-1β; (**B**): IL-6; (**C**): TNF-α.

To evaluate the effects of different immunization protocols on the respiratory inflammatory microenvironment in swine, the levels of the core pro-inflammatory cytokines IL-1β, IL-6, and TNF-α in BALF were further measured (Figure 4). The results showed that the levels of IL-1β, IL-6, and TNF-α in BALF of both the inactivated vaccine group and the live attenuated vaccine group were significantly lower than those in the non-vaccinated control group (*p* < 0.01). Among the groups, the non-vaccinated control group had the highest levels of the three inflammatory cytokines, indicating a high level of respiratory inflammation in unvaccinated swine. After vaccination, the levels of respiratory inflammation were markedly reduced, and the live attenuated vaccine group exhibited the lowest levels of each inflammatory cytokine overall. These results suggest that Mhp vaccination effectively and differentially regulates the inflammatory response level in the porcine respiratory tract.

### 3.5. Lung Lesion Scores for Different Immunization Protocols

**Figure 5:**
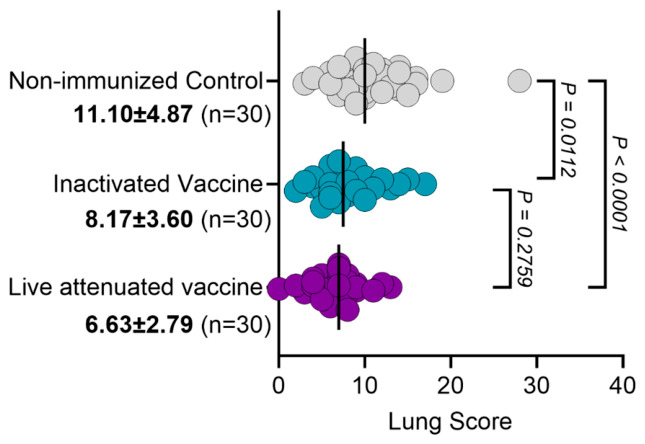
Gross lung lesion scores.

The lung pathology scoring results (Figure 5) showed that the non-vaccinated control group had the highest lung lesion score (11.10 ± 4.87), indicating that Mhp infection caused relatively severe pulmonary pathological damage in the swine. Compared with the non-vaccinated control group, both the inactivated vaccine group (8.17 ± 3.60) and the live attenuated vaccine group (6.63 ± 2.79) exhibited significantly lower lung scores, confirming that Mhp vaccination effectively reduces Mhp-induced lung tissue damage.

These results indicate that Mhp immunization significantly reduces the impact on porcine lungs, and the tissue protective effect of the live attenuated vaccine is superior to that of the inactivated vaccine.

### 3.6. Effects of Different Immunization Protocols on Pulmonary Histopathology

**Figure 6:**
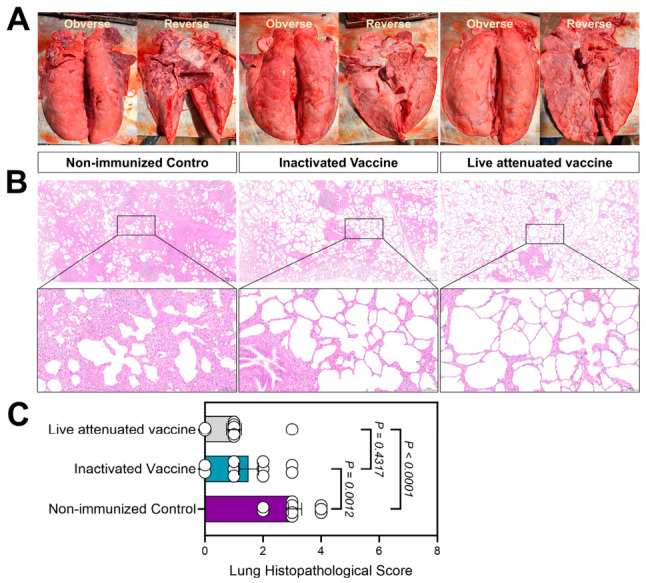
Effects of different immunization protocols on pulmonary histopathology. (**A**): Representative gross anatomy of porcine lungs (Obverse: ventral view; Reverse: dorsal view); (**B**): H&E-stained sections of porcine lung tissue (upper panel: 40×; lower panel: 200×); (**C**): Statistical results of porcine pulmonary histopathological scores.

Lung necropsy and H&E staining were performed to evaluate the effects of different immunization protocols on porcine pulmonary histopathological changes. Gross lung dissection results (Figure 6A) showed that the lungs of swine in the non-vaccinated control group exhibited numerous dark red parenchymal lesions, indicating substantial lung tissue damage induced by Mhp infection. In contrast, lung lesions were significantly alleviated and parenchymal lesions were markedly reduced in the vaccinated groups. Histological observations by H&E staining (Figure 6B) further confirmed that the non-vaccinated group showed severe inflammatory cell infiltration, alveolar structure destruction, and peribronchial inflammation. The inactivated vaccine group exhibited markedly reduced inflammatory infiltration and largely intact alveolar structures. The live attenuated vaccine group showed clear alveolar structures with only sporadic, focal inflammatory cell infiltration, representing the most pronounced improvement in lesions. Pulmonary histopathological scoring results (Figure 6C) showed that compared with the non-immunized control group, both vaccine-immunized groups had significantly lower lung lesion score (*p* < 0.01). Although the live attenuated vaccine group showed numerically lower histopathological scores than the inactivated vaccine group, the difference was not statistically significant (*p* = 0.4317).

Together, these results indicate that both vaccines effectively alleviate Mhp infection-induced lung consolidation, necrosis, and inflammatory cell infiltration, with the live attenuated vaccine showing numerically superior tissue protection.

### 3.7. Effects of Different Immunization Protocols on Swine Production Performance and Economic Indicators

To systematically evaluate the effects of different immunization protocols on production performance and economic indicators, all relevant parameters were recorded in detail by the farm’s marketing department (Table 6). Due to the constraints of commercial rearing and sales practices, swine were sold in groups without individual weighing; therefore, all production and economic indicators are presented as group means (calculated as total group weight divided by the number of swine sold).

In terms of production performance, compared with the non-immunized group, both vaccinated groups showed reduced mortality and feed conversion ratio, with the live attenuated vaccine group exhibiting a greater reduction. Regarding average daily gain, the live attenuated vaccine group showed a marked increase compared with the non-immunized group, whereas the inactivated vaccine group showed a decrease. Additionally, the inactivated vaccine group required 5 more days to reach the market weight (120 kg) than the non-immunized group (177 vs. 172 d), while the live attenuated vaccine group required 3 fewer days than the non-immunized group (169 vs. 172 d). Compared with the inactivated vaccine group, the live attenuated vaccine group had an 8-day shorter rearing period (169 vs. 177 d). This may be related to the later immunization age and failure to effectively induce mucosal immunity in the inactivated vaccine group, leading to early subclinical infection that consumed some growth potential.

In terms of economic benefits, the combined vaccination and medication cost per swine in both vaccinated groups was lower than that in the non-immunized group (49.09 CNY), with the inactivated vaccine group at 42.44 CNY (a reduction of 6.65 CNY) and the live attenuated vaccine group at 31.17 CNY (a reduction of 17.92 CNY), which was 11.27 CNY lower than that of the inactivated vaccine group.

These results indicate that immunization with the Mhp live attenuated vaccine effectively improves average daily gain, reduces mortality and feed conversion ratio, while shortening the rearing period and reducing the combined vaccination and medication cost per swine.

## 4. Discussion

Although various strategies, including vaccination, have been employed to control Mhp infection, the success of disease eradication in swine farms worldwide has not reached an ideal level [17,22]. In particular, in regions with intensive swine production, the elimination of Mhp remains a critical challenge that the swine industry urgently needs to address [23]. In the present study, a baseline survey of the production area where the trial was conducted revealed an Mhp positivity rate as high as 64%, far exceeding the previously reported average level of 48.09% in Guangxi [9], indicating that this farm faced extremely high field challenge pressure. A high positivity rate implies that the herd is under constant risk of infection, which may not only directly affect growth performance but also increase the risk of secondary infections with other pathogens. Therefore, under such high-infection-pressure conditions, the selection of a vaccine that can provide early and comprehensive protection is particularly critical for integrated MPS control. In this study, under actual field conditions in a large-scale swine farm, the application value of inactivated and live attenuated MPS vaccines was systematically compared in terms of clinical control efficacy, improvement of pathological damage, and production economic benefits.

The present trial confirmed that nasal and throat swabs show high sensitivity and consistency for Mhp antigen detection, with results being highly concordant between the two sampling methods. This finding suggests that for large-scale pathogen surveillance under field conditions, nasal swab sampling is preferable as the first choice method due to its ease of operation, minimal animal stress, and higher compliance [24], thereby greatly improving the efficiency of on-farm disease monitoring. Clinical safety evaluation of vaccines is a prerequisite for conducting comparative studies on immune efficacy [17,23]. Although inactivated vaccines fail to effectively induce local respiratory mucosal immunity [25], they are widely used in swine production due to high systemic safety and minimal stress responses [26]. The safety monitoring results of this trial showed that no systemic or local adverse reactions occurred in swine after immunization with either vaccine, indicating that both vaccines exhibit good safety for field application and provide a reliable foundation for subsequent efficacy evaluations.

Mhp-specific IgG antibodies in swine can be induced either by natural infection or by vaccination [20]; relying solely on serum IgG detection cannot distinguish immune status from infection status and may easily lead to misdiagnosis. In this study, all experimental piglets were confirmed as Mhp antigen-negative, thereby effectively ruling out interference from fieldinfection on the evaluation of the above indicators. On this basis, the present study adopted a combined detection scheme using nasal swab sIgA-ELISA for mucosal antibodies and serum IgG-ELISA for systemic antibodies to cross-validate the results [24]. The antibody monitoring results showed that the live attenuated vaccine significantly increased specific sIgA antibody levels in nasal swabs, whereas the inactivated vaccine failed to induce a significant local sIgA immune response; both vaccines significantly elevated serum IgG antibody levels. The advantage of the live attenuated vaccine lies in its ability to effectively colonize and replicate locally in the respiratory tract, providing sustained antigenic stimulation and thereby concurrently enhancing both local mucosal immunity and systemic long-lasting humoral immune protection [25]. With respect to pulmonary inflammatory regulation and histopathology, the non-immunized control group exhibited the highest levels of inflammatory cytokines in BALF, as well as the most severe gross lung lesion scores and histopathological damage, further confirming that Mhp infection induces a robust inflammatory response in the host [4]. After immunization with either vaccine, the levels of inflammatory cytokines decreased significantly, and lung lesions were markedly alleviated, indicating that vaccination effectively suppresses the excessive inflammatory response triggered by Mhp. Among the two vaccine groups, the live attenuated vaccine group showed overall lower inflammatory cytokine levels than the inactivated vaccine group, as well as better gross lung lesion scores and histopathological scores. Although the histopathological scores were numerically lower in the live attenuated vaccine group compared with the inactivated vaccine group, the difference was not statistically significant (*p* = 0.4317), likely due to the limited sample size (*n* = 30 per group) and insufficient statistical power. The numerical trend, however, was consistent with the improvements observed in inflammatory cytokine levels and gross lung lesions. In addition, the live attenuated vaccine in this trial was administered at 7 days of age. Compared with the conventional immunization schedule at 14 days of age, earlier establishment of immune protection may be another important factor contributing to its superior performance. Nevertheless, it should be acknowledged that the two vaccines differed in both administration route and vaccination age, as each was used according to its respective commercial label recommendations. Therefore, the observed differences cannot be attributed solely to vaccine type, and these confounding factors should be considered when interpreting the results.

Production performance and economic benefits are the ultimate indicators for evaluating the field application value of vaccines. Previous studies have shown that Mhp infection can reduce the profit per finishing swine by approximately €5 [27]. Such economic losses mainly stem from growth delay, an increased feed conversion ratio, and higher costs associated with drugs used for disease control [10,28]. Early immunization to reduce disease incidence substantially decreases the use of antimicrobials and hormones, which is of great significance for both animal health and public health safety [22]. The results of this study showed that the live attenuated vaccine group achieved superior production performance, with a three-day shorter finishing period compared with the inactivated vaccine group, and exhibited the lowest cost of vaccines and drugs per swine, demonstrating significant economic value for swine production. However, we acknowledge that the economic assessment in this study was limited to direct vaccine and medication costs, and did not include other potential economic indicators such as labor costs, carcass quality, or treatment costs for secondary infections.

Nevertheless, this study has certain limitations. Under field trial conditions, it is impossible to completely exclude interference from external environmental factors and other pathogens. Although zone-based rearing and enhanced biosecurity measures were implemented, potential co-infections may still have influenced the results. Additionally, farm management level is also a key factor affecting vaccine immune efficacy, and its potential role has not yet been fully elucidated. Furthermore, as the trial was conducted on a single farm, the generalizability of the findings to other farms with different management practices, genetic backgrounds, or Mhp strain profiles requires further validation through multi-site studies. Moreover, cellular immune responses (such as T lymphocyte subsets, IFN-γ, and lymphocyte proliferation levels) were not evaluated in this study, which represents a limitation in fully characterizing the immune mechanisms underlying the two vaccines. Future studies should focus on determining the optimal vaccination timing for live attenuated vaccine administration by comparing different age windows and evaluating the need for booster immunization strategies. In addition, the compatibility and combined immunization protocols with other commonly used swine vaccines should be systematically investigated to facilitate field implementation. Finally, multi-site field trials under different Mhp infection pressure gradients, management practices, genetic backgrounds, and circulating Mhp strain profiles are needed to validate the generalizability of the findings and clarify the economic applicability of the live attenuated vaccine under diverse farm conditions.

## 5. Conclusions

Under the conditions of this single-farm trial, the live attenuated Mhp vaccine (administered intrapulmonarily at 7 days of age) showed superior outcomes in mucosal immunity, pulmonary protection, production performance, and economic benefits compared with the inactivated vaccine (administered intramuscularly at 14 days of age). However, because the two vaccines differed in both administration route and vaccination age, the observed superiority cannot be attributed solely to vaccine type. Multi-farm studies with standardized protocols are needed before broader conclusions can be drawn.

## Figures and Tables

**Table 1:** Primer sequence.

Primer Names	Sequences (5′ to 3′)
Mhp-183-F	CAAAGCGAGTATGAAGAACAAGAAA
Mhp-183-R	GTCATCATTGGGTGGCTAAGT
Mhp-183-P (probe)	ROX-TCCAGGAAGTCAAGGTAACTAGTGACCA-BHQ

**Table 2:** Scoring criteria for safety evaluation.

Parameter Description		Score
Activity	Active	3
	Depressed	2
	Extremely listless	1
Abnormal behavior	No abnormality	4
	Occasional cough	3
	Frequent cough, labored breathing	2
	Persistent cough, dyspnea	1
Feed and water intake	Normal	3
	Reduced	2
	Refusal	1
Body temperature	Normal	3
	Slight change	2
	Markedly abnormal	1
Local reaction at injection site	No abnormality	2
	Abnormal	1

**Table 3:** Scoring criteria for lung lesions (28-point system).

Score	Percentage of Surface Affected and Judgment Basis
0	0%—no lesion
1	1–25% surface affected
2	26–49% surface affected
3	50–74% surface affected
4	≥75% surface affected

Note: All scores were assigned by visual inspection.

**Table 4:** Scoring criteria for H&E.

Score	Microscopic Findings
0	Normal
1	Mild inflammatory cell infiltration
2	Moderate focal inflammatory cell infiltration
3	Moderate diffuse inflammatory cell infiltration
4	Severe diffuse inflammatory cell infiltration

**Table 5:** Mhp positivity rate in swine herds.

Sampling Method	Number of Swine in Herd	Number Tested	Number Positive	Positive Rate (%)
Nasal swabs	3500	350	224	64.00
Throat swabs	3500	350	224	64.00

**Table 6:** Comparison of production performance and economic indicators among different groups.

Group	ASW (kg)	ADG (g)	MR (%)	FCR	Rearing Period (d)	Cost/Swine (CNY)
Non-vaccinated	120.45	666.8	4.06	3.01	172	49.09
Inactivated vaccine	121.08	649.9	3.56	2.97	177	42.44
Live attenuated vaccine	125.34	704.1	3.32	2.77	169	31.17

Note: ASW: Average sale weight; ADG: average daily gain; MR: mortality rate; FCR: feed conversion ratio.

## Data Availability

The data that support the findings of this study are available from the corresponding author upon reasonable request (jkhe@gxu.edu.cn).

## Abbreviations

The following abbreviations are used in this manuscript:

ADG	Average daily gain
ASW	Average sale weight
BALF	Bronchoalveolar lavage fluid
FCR	Feed conversion ratio
H&E	Hematoxylin and eosin
Mhp	*Mycoplasma hyopneumoniae*
MPS	*Mycoplasmal pneumonia* of swine
MR	Mortality rate
sIgA	Secretory immunoglobulin A
TNF-α	Tumor necrosis factor alpha
